# Heard but not seen: Comparing bat assemblages and study methods in a mosaic landscape in the Western Ghats of India

**DOI:** 10.1002/ece3.3942

**Published:** 2018-03-23

**Authors:** Claire F. R. Wordley, Mahesh Sankaran, Divya Mudappa, John D. Altringham

**Affiliations:** ^1^ School of Biology University of Leeds Leeds UK; ^2^ National Centre for Biological Sciences Tata Institute of Fundamental Research, GKVK Bangalore India; ^3^ Nature Conservation Foundation Mysore India

**Keywords:** bats, coffee plantations, echolocation, forest fragmentation, riparian corridors, tea plantations

## Abstract

We used capture (mist‐netting) and acoustic methods to compare the species richness, abundance, and composition of a bat assemblage in different habitats in the Western Ghats of India. In the tropics, catching bats has been more commonly used as a survey method than acoustic recordings. In our study, acoustic methods based on recording echolocation calls detected greater bat activity and more species than mist‐netting. However, some species were detected more frequently or exclusively by capture. Ideally, the two methods should be used together to compensate for the biases in each. Using combined capture and acoustic data, we found that protected forests, forest fragments, and shade coffee plantations hosted similar and diverse species assemblages, although some species were recorded more frequently in protected forests. Tea plantations contained very few species from the overall bat assemblage. In riparian habitats, a strip of forested habitat on the river bank improved the habitat for bats compared to rivers with tea planted up to each bank. Our results show that shade coffee plantations are better bat habitat than tea plantations in biodiversity hotspots. However, if tea is to be the dominant land use, forest fragments and riparian corridors can improve the landscape considerably for bats. We encourage coffee growers to retain traditional plantations with mature native trees, rather than reverting to sun grown coffee or coffee shaded by a few species of timber trees.

## 1 INTRODUCTION

Bats are an understudied taxon in the palaeotropics, despite their high diversity, important ecosystem roles, and value as bio‐indicators (Jones, Jacobs, Kunz, Willig, & Racey, 2009; Kunz, de Torrez, Bauer, Lobova, & Fleming, 2011). About 25% of bat species are considered to be threatened (Jones, Purvis, & Gittleman, 2003), but with limited data on how palaeotropical bat species respond to different land uses it is difficult to anticipate which species are most at risk of decline from habitat conversion (Kingston, 2010; Meyer, Struebig, & Willig, 2016). This lack of data is further complicated by the inherent but often unknown biases in the methods used to study bats. While in temperate regions, almost all studies have an acoustic component, where the echolocation calls of the bats are recorded and identified, in the tropics, especially the palaeotropics, most studies have relied exclusively on capturing bats in mist‐nets, or, more recently, harp traps (Meyer et al., 2016). This difference is driven by the initial outlay costs of acoustic equipment, the lack of bat echolocation call libraries in the tropics and the difficulty of identifying bat calls in species‐rich assemblages. In this study, we explore the impacts of land‐use change on bats in south India, and the relative merits of two widely used techniques for surveying bats.

All methods for studying bats have potential advantages and disadvantages. Catching bats often allows better species‐level identification than acoustic methods and allows the collection of data on biometrics, sex and reproductive status, and genetic material. However, it is time‐consuming, invasive, and can lead to bias as the percentage of the airspace sampled is small and usually close to the ground, and some species are better at avoiding capture than others (O'Farrell & Gannon, 1999).

These drawbacks have been compensated for in recent studies by the use of ultrasound detectors, which detect the echolocation calls of bats. Acoustic recordings can help achieve a more complete species list for the area, and sample some species that are never caught (Kalko, Handley, Handley, Handley, & Handley, 1996; MacSwiney, Clarke, & Racey, 2008). However, some species cannot be separated using echolocation calls alone, low intensity echolocators and nonecholocating bats are under‐sampled or not sampled at all (O'Farrell & Gannon, 1999), and ultrasound does not travel far in dense vegetation. Several studies indicate that combining acoustic and capture data give the most complete picture of the bat assemblage (Furey, Mackie, & Racey, 2009; MacSwiney et al., 2008; Murray, Britzke, Hadley, & Robbins, 1999; O'Farrell & Gannon, 1999), yet ecological studies of bats generally report data gathered using only one method.

In this study, we compare and combine results from capture (mist‐netting) and acoustic surveys of a palaeotropical bat assemblage to assess the responses of bats to agricultural land uses in South Asia. While harp trapping is widely used in South‐East Asia and offers some advantages over mist‐netting (Kingston, 2013), we chose to use mist‐nets in this study as they are more widely available, easier to transport and deploy in dense understorey, can cover much larger airspaces per unit cost, and are the capture method most commonly used in India. Our early efforts to use 4.2 m^2^ two‐bank harp traps to catch in forest fragments and coffee plantations were unsuccessful despite high numbers of bats captured in tunnels using these traps (Wordley, Foui, Mudappa, Sankaran, & Altringham, 2014), and we found it difficult to set the traps up in dense understorey, so we reverted to using only mist‐nets.

We employed both mist‐netting and acoustic sampling in seven habitats (tea (*Camellia sinensis*) plantations, shade coffee (*Coffea arabica* and *C. canephora*) plantations, forest fragments, protected forest, rivers in tea, rivers in tea with riparian corridors, and rivers in protected forests). There were insufficient suitable rivers with coffee planted up to each bank to study bat assemblages in this habitat. We predicted that acoustic sampling would record more insectivorous species, but that mist‐netting would capture more frugivorous species, across all habitats. We predicted that nonecholocating frugivorous species would be detected by capture alone, but that all other species would be more frequently detected by acoustic sampling. We then used these data to compare the relative bat diversity in each habitat.

Despite its size, and the fact that it hosts 10% of the world's bat species, there have been few ecological studies of India's bats. The Western Ghats are, together with Sri Lanka, the eighth “hottest” biodiversity hotspot in the world (Myers, Mittermeier, Mittermeier, da Fonseca, & Kent, 2000), yet only 6% of the land area of the Western Ghats remains under primary vegetation (Sloan, Jenkins, Joppa, Gaveau, & Laurance, 2014). Most of the remaining forest survives as small fragments in a matrix of agricultural land including coffee and tea plantations (Menon & Bawa, 1997). Since 2000, the Nature Conservation Foundation (NCF) has been working to extend and restore the forest fragments in our study area, the Anamalai Hills around Valaparai, and to encourage local coffee growers to shade their coffee with native shade trees rather than commercial timber trees (Mudappa & Raman, 2007). NCF has also been working to understand the relative diversity of different taxa from spiders to mammals in protected forests, forest fragments, and different types of plantations (Kapoor, 2008; Kumar, Mudappa, & Raman, 2010).

Assessment of the value of agroforestry plantations for bats in the palaeotropics has been identified as a key research need (Meyer et al., 2016). Globally, there was a 20% decline in shade‐grown agroforestry coffee between 1996 and 2010, such that only 24% of coffee is now managed under diverse shade (Jha et al., 2014). Neotropical studies show that coffee and cacao grown under a canopy of native shade trees can provide a good habitat for many bat species (Faria, Laps, Baumgarten, & Cetra, 2006; Harvey & Villalobos, 2007; Pardini et al., 2009; Pineda, Moreno, Escobar, & Halffter, 2005; Williams‐Guillén & Perfecto, 2010, 2011). The few studies on bat diversity in coffee from Asia give similar results (Graf, 2010; Wordley, Sankaran, Mudappa, & Altringham, 2015, 2017); however, little is known about the value of this habitat for most palaeotropical bat species.

Studies on palaeotropical bats in a range of agricultural land uses, especially large‐scale commercial uses, have also been identified as a key research need (Meyer et al., 2016). Tea is a widespread and expanding commercial land use across the palaeotropics (FAOSTAT 2014). In the study site, as is typical, it is grown as clipped bushes with light shade from Australian silver oak trees (*Grevillea robusta*). Wordley et al. (2015) demonstrated that many bat species avoided areas with a high coverage of tea plantations, and similar patterns have been documented for birds and frogs (Murali & Raman, 2012; Sidhu, Raman, & Goodale, 2010). As climate change is likely to lead to an upslope expansion of the areas suitable for tea and coffee cultivation globally, it is important to understand the likely relative impacts of these two plantation types on biodiversity.

Riparian habitats are important foraging areas for many bat species, due to the abundance of insects. Studies have found that bankside vegetation significantly increased bat activity over rivers, but these have mostly been from temperate regions (Lundy & Montgomery, 2009; Ober, Hayes, & Hall, 2008; Warren, Waters, Altringham, & Bullock, 2000). While Indian law does not currently legislate to promote riparian corridors of vegetation along river banks, in other countries riparian corridors are compulsory in certain land uses, particularly heavily modified plantations, to reduce erosion of river banks, intercept fertilisers, and provide habitats for biodiversity (Marczak et al., 2010; Mayer, Reynolds, McCutchen, & Canfield, 2007; Sweeney et al., 2004).

By studying bat species in a range of habitats in the Valparai plateau and adjacent Anamalai Tiger Reserve, we aim to determine which species survive in human‐modified landscapes, and which decline or disappear. We also aim to determine the relative contribution made by human‐modified habitats such as fragmented forests, agroforestry plantations, and monoculture plantations to maintaining bat diversity, and which methods are most appropriate for measuring this diversity. We predict that fragmented forests will have lower diversity and altered species composition compared to protected forests; that shade‐grown agroforestry coffee plantations will retain a similar but less diverse bat assemblage compared to forest fragments; and that tea plantations will have the lowest diversity of all. We predict that the presence of riparian corridors on rivers in tea plantations will increase the bat diversity on those rivers compared to rivers without riparian corridors, but that rivers in protected areas will retain the highest diversity. We predict that forest‐adapted species such as Megadermatidae, Rhinolophidae, and Hipposideridae will show the greatest declines in all nonprotected habitats, and that fruit bats will be largely absent from tea plantations.

## 2 MATERIALS AND METHODS

### 2.1 Study area

This study was conducted on the Valparai plateau and adjacent Anamalai Tiger Reserve in the state of Tamil Nadu in the southern Western Ghats (N 10.2–10.4°, E 76.8–77.0°). The Valparai plateau is an agricultural landscape approximately 800–1,600 m asl dominated by tea plantations interspersed with shade‐grown coffee plantations, eucalyptus plantations, rainforest fragments, streams, and riparian vegetation (Mudappa & Raman, 2007). Forest fragments and riparian corridors were remnant forest patches or secondary forest/overgrown plantations dominated by mature native trees. Several of these fragments have received supplementary planting to restore and extend them (Mudappa & Raman, 2007). The native vegetation is mid‐elevation tropical wet evergreen forest of the *Cullenia exarillata–Mesua ferrea–Palaquium ellipticum* type (Pascal, 1988; Raman, Mudappa, & Kapoor, 2009). For a detailed map of the study area, see Wordley et al. (2015). The average annual rainfall is 3,500 mm, of which about 70% falls during the southwest monsoon (June–September; Raman et al., 2009).

### 2.2 Data collection

We chose five sites for each of the seven study habitats, and between January and May 2010 to 2013, and in November–December 2014, we spent a total of two nonconsecutive nights at each site capturing bats and recording echolocation calls. We caught bats and recorded them on the same night to reduce the effects of inter‐night variation. At every site, we caught bats using five ground level (6 m x 2.5 m) mist‐nets 50–200 m from the nearest acoustic sampling point, and recorded at five points 100 m apart for 15 min per point every night. We started recording 40 min after sunset as bats begin foraging at different times relative to sunset, so until it is fully dark, each acoustic recording point may be subject to temporal bias. We used a handheld Pettersson D240X ultrasound detector (http://www.batsound.com) recording onto an Edirol R‐09 (http://www.roland.com) digital recorder. The detector was set to constantly sample, so a trigger level was not used. The detector was moved in a semicircular arc to record bats from a wider section of the aerospace. Nets were opened at sunset and closed after 2.5 hr. Bats caught in nets were identified to species using Bates and Harrison (1997) and Srinivasulu, Racey, and Mistry (2010). In riparian habitats, all nets were set over the river, and recordings were made on the river banks, pointing at the river, so only species close to the river would be recorded. All rivers were at least 4 m wide at the point of sampling. Forest fragment size varied from 2.2 to 102.8 ha, riparian corridor area from 3.7 to 159.7 ha, and riparian corridor width from 17 to 1,070 m at the widest point. All study sites were at least 1 km apart, and spatial auto‐correlation of bat species presence was low (Wordley et al., 2015) so has not been considered here. The elevation range in this study did not affect the likelihood of the presence of any of the species modeled by Wordley et al. (2015), so has not been considered here.

### 2.3 Sound analysis

Echolocation calls were visualized as spectrograms to measure call parameters using BatSound (http://www.batsound.com). Calls were manually identified using an echolocation call library for the area (Wordley et al., 2014). At each recording point, a species was marked as present if a call unambiguously attributable to that species was recorded within the 15 min recording. Due to call overlap between species (Wordley et al., 2014), not all species were easily identifiable. *Scotophilus heathii* and *Pipistrellus ceylonicus* overlapped extensively in call frequency and had the same call structure, but *S. heathii* calls were clustered toward the higher end of the *P. ceylonicus* range with only one call as low as 33.8 kHz; here we have classified calls of 31–34 kHz as *P. ceylonicus*, and not attempted to identify frequency modulated (FM) calls with a quasi‐constant frequency (QCF) tail between 34 and 44 kHz, meaning that *S. heathii* is not represented in acoustic data. Even though the end frequencies and FMAXE (Frequency of Maximum Energy) overlapped, in practice *Myotis horsfieldii* and *Miniopterus fuliginosus* calls could be easily identified by differing call structure (FM in the former and FM with a QCF tail in the latter). The calls of *Scotophilus kuhlii* and *Myotis peytoni* (previously *M. montivagus*) were, however, difficult to distinguish from *M. fuliginosus*. As some *M. fuliginosus* and all *S. kuhlii* calls had end frequencies under 45 kHz, we ignored calls of end frequency 40–44 kHz and classed frequency modulated calls with a quasi‐constant frequency tail of 45–53 kHz as *M. fuliginosus*, meaning that *M. peytoni* and *S. kuhlii* are not represented in acoustic data. *M. peytoni* calls were difficult to tell apart in practice from *M. fuliginosus* so we cannot be sure that there are not some *M. peytoni* calls misclassified as *M. fuliginosus*. However, given the apparent scarcity of *M. peytoni*—we caught three *M. peytoni* compared to 78 *M. fuliginosus* and 71 *M. horsfieldii*—this is unlikely to add many false positive data points. As globally there is only a recording from a single *Hesperoptenus tickelli* (Wordley et al., 2014), we only classified bats falling within the frequency range seen in this individual recording (18–22 kHz) *as H. tickelli*, meaning this species may be underrepresented in acoustic recordings. The assemblage had only two nonecholocating species (*Cynopterus brachyotis* and *Latidens salimalii*), the latter being excluded from analysis as it was never recorded more than once per habitat.

Echolocation calls that we could not identify to species were removed from all further analyses, along with per habitat singletons which are likely to be functionally unimportant in the assemblage (McConkey & O'Farrill, 2015).

### 2.4 Species rarefaction curves

We generated individual‐based species rarefaction curves combining capture and acoustic data for each habitat, using the R packages “picante” and “vegan,” using the formula “rarefaction” (http://www.jennajacobs.org/R/rarefaction.html; Kembel et al., 2010; Oksanen et al., 2013; R Core Team, 2014). We calculated the “Chao” estimated species richness per habitat and per site for each method using the “vegan” package in R.

### 2.5 Species richness

We combined the data from both nights at each site to avoid pseudo‐replication. Generalized linear mixed models would not converge for these data, so we ran a Poisson generalized linear model (GLM) in “lme4” with method (capture, acoustic sampling) and habitat as the predictor variables, and compared models with and without each factor to the full model using a likelihood ratio χ^2^ test (Bates, Maechler, Bolker, & Walker, 2014). We ran pairwise comparisons and corrected for multiple testing using the false discovery rate (FDR) method in the “lsmeans” package in R (Lenth, 2014).

### 2.6 Size effects

Areas of forest fragments and riparian corridors were calculated using ArcGIS (Wordley et al., 2015). Riparian corridor width (perpendicular to river bank) was measured at each acoustic transect point and the mean taken per corridor. Linear regression analyses were performed in R to look at the effects of forest fragment area, riparian corridor area, and riparian corridor width on bat species richness (Table S7).

### 2.7 Activity

The total number of “records” of bats per method and per site were counted. We counted every bat captured as one record, and every species recorded in a 15‐min acoustic recording as a record. We did not count the number of calls per species at each point, to reduce bias from recording the same individual bat multiple times or due to different likelihood of detection of different species. We followed the same procedure as for species richness except that we ran a quasi‐Poisson GLM due to over‐dispersion.

### 2.8 Species composition

Using the “PERMANOVA” (permutational multivariate analysis of variance using distance matrices) method executed through the “ADONIS” function in “vegan” with 9,999 permutations, we tested for differences in species composition between habitats, and ran pairwise comparisons using FDR.

For each species with >30 records in total, we used Kruskal–Wallis tests on site level data to test for changes in the activity between habitats, using the “agricolae” package in R to conduct pairwise comparisons with FDR correction (de Mendiburu, 2014).

## 3 RESULTS

### 3.1 Species richness

**Figure 1:**
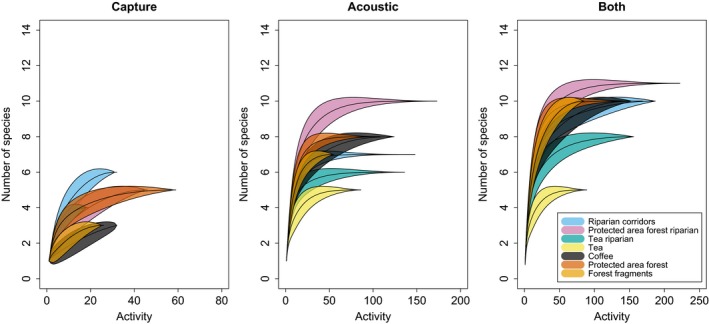
Species rarefaction curves with 95% confidence intervals per habitat for capture data, acoustic data, and acoustic and capture data combined

We recorded 17 species (Table 1). Observed species richness was equal or close to the estimated species richness per habitat and per site when both detection methods were combined (Figure 1, Table S1). When capture data alone were used, species richness and estimated species richness were considerably lower, with no bats captured in tea and higher species richness in tea riparian and riparian corridors than in protected area forest. When acoustic data alone were used, the estimated species richness was slightly lower across the board than when methods were combined, but the overall pattern was very similar; except in forest fragments where estimated species richness was five compared to 8.5 when methods were combined (Table S1). Combined capture and acoustic data and acoustic data alone yielded significantly higher species richness than capture data alone, for all habitats, but not significantly higher than for acoustic data alone (Table S2).

**Table 1:** Total numbers of each species captured or recorded in each habitat (per habitat singletons removed in analysis)

Species	Protected area forest	Forest fragments	Coffee	Tea	Protected area forest river	Riparian corridor	Tea riparian
*Cynopterus brachyotis*	C: 22	22	29		31	13	
	A:						
*Hesperoptenus tickelli*	C:						
	A: 1	1	4	1	6	1	
*Hipposideros pomona*	C:	1		1			
	A: 6	1	3		5	1	1
*Latidens salimalii*	C:				1	1	
	A:						
*Megaderma spasma*	C: 9	2					
	A:						
*Miniopterus fuliginosus*	C:				2	3	2
	A: 11	8	29	32	21	25	28
*Miniopterus pusillus*	C:				9	1	
	A: 27	5	20	8	26	11	9
*Myotis horsfieldii*	C:				2	3	2
	A:				26	27	26
*Myotis peytoni*	C:					2	
	A:						
*Pipistrellus ceylonicus*	C:				1	10	8
	A: 21	20	36	38	35	42	47
*Rousettus leschenaultii*	C:		1	1		1	2
	A:					6	
*Rhinolophus beddomei*	C:		2		3	2	
	A: 13	2			10	1	
*Rhinolophus indorouxii*	C: 1	2					
	A: 4	6	9	3	4	14	4
*Rhinolophus lepidus*	C: 2	1			2	2	
	A: 15	11	21	5	32	23	22
*Rhinolophus rouxii*	C: 23				1		
	A: 11	3	2		8		
*Scotophilus heathii*	C:						7
	A:						
*Scotophilus kuhlii*	C:					2	
	A:						

C, Capture; A, Acoustic.

**Figure 2:**
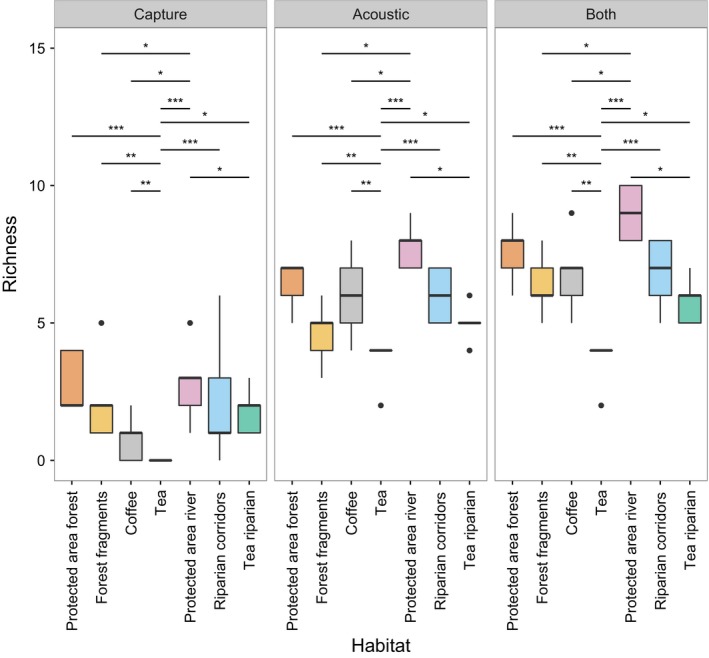
Species richness by habitat and method, shown as boxplots with quartiles, whiskers extending to 1.5 times the interquartile range of the nearest hinge, and outliers as points. Stars indicate significance: *p ≤ .05, **p ≤ .01, ***p ≤ .001

Species richness differed between habitats (deviance = 773.7, *df* = 7, *p *<* *.001) and with the sampling methods used (deviance = 850.9, *df* = 3, *p *<* *.001), but there was no significant interaction (deviance = 16.47, *df* = 12, *p *=* *.17). Species richness was significantly lower in tea plantations than in all other habitats (Figure 2) and highest in protected forest rivers followed by protected area forest. Rivers through protected area forest had significantly greater species richness than tea plantations, rivers through tea with no riparian corridor (hereafter tea riparian), coffee plantations, and forest fragments (Figure 2, Table S2).

Fragment area, riparian corridor area, and riparian corridor width did not have significant effects on the total species richness (*F*_1,3_ = 0.002, adjusted *R* ^2^ = −.333, *p* = .969; *F*_1,3_ = 0.117, adjusted *R* ^2^ = −.283, *p* = .755; *F*_1,3_ = 0.164, adjusted *R* ^2^ = −.264, *p* = .713, respectively; Table S7).

### 3.2 Activity

**Figure 3:**
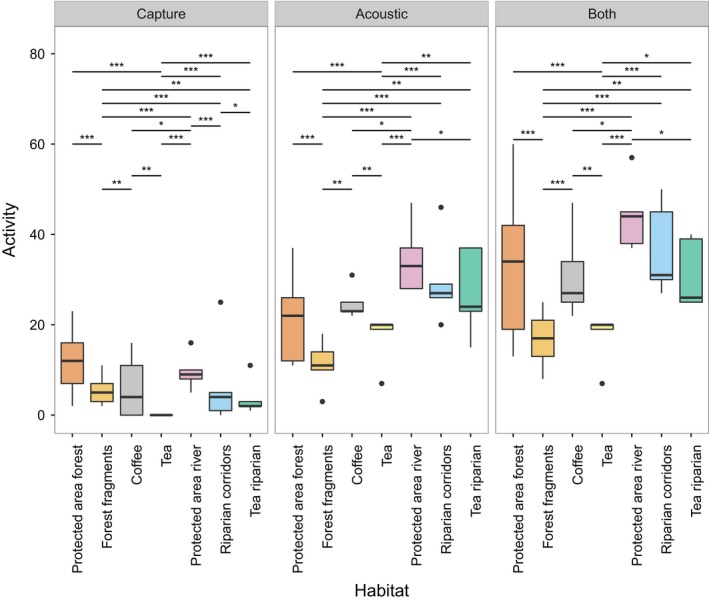
Activity of bats by habitat and method, boxplots and significance stars shown as boxplots with quartiles, whiskers extending to 1.5 times the interquartile range of the nearest hinge and outliers as points. Stars indicate significance: *p ≤ .05, **p ≤ .01, ***p ≤ .001. Activity refers to number of individuals caught for capture, and number of species per recording for acoustic (Section 2.7)

Habitat had a significant effect on overall activity (deviance = −2,286.02, *df* = −18, *p *<* *.001), as did method (deviance = −699.88, *df* = −14, *p *<* *.001), but the interaction was not significant (deviance = −75.26, *df* = −12, *p *=* *.074). Activity was significantly lower in tea plantations and forest fragments than in all other habitats when methods were combined, but with capture data alone, forest fragments had slightly higher activity than coffee plantations (Figure 3, Table S3). Activity using combined data was significantly higher on rivers in protected area forest than in all other habitats except riparian corridors. Activity was significantly lower for capture data than for combined data, whereas activity for acoustic data was not significantly different from combined.

### 3.3 Species composition

Species composition (using combined acoustic and capture data) differed significantly between habitats (Table 2). Protected area forests were significantly different in species composition from all riparian habitats and tea plantations, but not coffee plantations or forest fragments. Coffee and forest fragments did however differ significantly from each other in species composition. Rivers in protected area forest were significantly different from riparian corridors and tea riparian, as well as all the nonriparian habitats. Tea plantations were significantly different in species composition from all other habitats. Several species showed significant changes in activity between habitats (Table S4).

**Table 2:** Species composition (ADONIS) differences from combined capture and acoustic methods with false discovery rate (FDR) corrections . Stars indicate significance: *P*≤ 0.05 *, *P*≤ 0.01 **, *P*≤ 0.001 ***

Compare	Against	*F*	*p* Value	*Q* value (after FDR correction)
Coffee	Forest fragments	3.404	<.001***	<.001***
Coffee	Riparian corridors	2.078	.117	.129
Coffee	Tea riparian	3.404	.008**	.014*
Coffee	Tea	3.163	.009**	.014*
Coffee	Protected area forest	1.957	.142	.149
Coffee	Protected area forest riparian	3.102	.007**	.014*
Forest fragments	Riparian corridors	5.920	<.001***	<.001***
Forest fragments	Tea riparian	8.919	<.001***	<.001***
Forest fragments	Tea	6.293	<.001***	<.001***
Forest fragments	Protected area forest	2.128	.104	.122
Forest fragments	Protected area forest riparian	5.736	.01**	.014*
Riparian corridors	Tea riparian	0.717	.659	.659
Riparian corridors	Tea	4.565	.008**	.014*
Riparian corridors	Protected area forest	4.284	<.001***	<.001***
Riparian corridors	Protected area forest riparian	3.645	.017*	.022*
Tea riparian	Tea	3.993	<.001***	<.001***
Tea riparian	Protected area forest	5.764	.01**	.014*
Tea riparian	Protected area forest riparian	6.803	.009**	.014*
Tea	Protected area forest	5.394	<.001***	<.001***
Tea	Protected area forest riparian	10.863	<.001***	<.001***
Protected area forest	Protected area forest riparian	1.872	.097	.120

When only capture data were used, fewer significant differences in species composition were seen between habitats (Table S5). While *F* and *p* values were typically lower using acoustic data only compared to combined data, most significant results remained (Table S6).

## 4 DISCUSSION

### 4.1 Comparison of methods

We demonstrate that the use of acoustic methods is feasible in India, and that while combining mist‐netting and acoustic data maximizes the number of species that will be detected, acoustic methods alone give broadly similar results in terms of richness and composition to those obtained by combining both methods. Despite the fact that several species could not be identified from acoustic recordings due to overlapping call structure, acoustic methods gave consistently higher estimates of species richness and activity than mist‐netting (Figures 1, 2, 3), and detected more significant differences in species composition (Table 2).

Very few bats were caught in tea plantations (none, after per habitat singletons were removed); however, several species were recorded in tea acoustically (Table 1). This is likely due to the difficulty of catching bats in wide open spaces (O'Farrell & Gannon, 1999; Patriquin, Hogberg, Chruszcz, Barclay, & Barclay, 2003). Conversely, we noted a relative decrease in the activity and species richness in forest fragments using acoustic methods as compared to mist‐netting. This may be due to the dense understory in forest fragments absorbing echolocation calls, unlike protected area forest which had a fuller canopy and consequently a less dense understory. We recommend that people undertaking acoustic surveys in heavily vegetated habitats undertake experiments to determine the effects of vegetation on reducing call detection.

We strongly encourage the creation of more bat echolocation call libraries from India, and from the palaeotropics more widely, and the expansion of acoustic sampling as a study technique in the tropics. While acoustic methods are more difficult to implement in species‐rich tropical assemblages than in temperate regions, studies have shown that species‐level classification is possible for up to 66% of bat calls even in mega‐diverse countries such as Mexico (Zamora‐Gutierrez et al., 2016). Acoustic studies are certainly possible in India, where the bat assemblage in each study site is likely to be less diverse than in the neotropics or South‐East Asia (Mendenhall, Karp, Meyer, Hadly, & Daily, 2014), and call libraries so far have not exceeded twenty species per site (Raghuram, Jain, & Balakrishnan, 2014; Wordley et al., 2014). More acoustic monitoring in the palaeotropics would improve our understanding of the distribution and ecology of bats, and help with conservation prioritization by identifying which species are rare, and what their habitat preferences are.

### 4.2 Comparison of plantations, fragments and forest

This study found broadly similar species richness and composition between bat assemblages in protected area forest, coffee plantations, and forest fragments, indicating that the original bat assemblage need not be lost in a modified landscape so long as sufficient forest fragments and/or coffee under native shade is retained. Richness and activity were greatest in protected forests, although this was not significant due to high variance between sites (Figures 2 and 3). The similarity between the richness of assemblages in protected forests and forest fragments echoes the findings of Mendenhall et al., 2014; who found that globally more studies showed the same bat species richness in forest fragments as compared to minimally disturbed forest than showed a reduced species richness in forest fragments. Meta‐analyses in the tropics have suggested that the impacts of land‐use change on bats are somewhat less severe than for other animal taxa (Gibson et al., 2011). However, species richness may mask changes in species composition and/or the occurrence of trait filtering, which may lead to the loss of some species and a lowering of functional diversity (Meyer et al., 2016; Struebig, Kingston, Zubaid, Mohd‐Adnan, & Rossiter, 2008; Villéger et al. 2008; Wordley et al., 2017).

There have been few generalizations about bats' responses to habitat fragmentation, and many responses appear highly species and guild specific (Meyer et al., 2016). Kingston (2010) and Meyer et al. (2016) identified a need for more work on the ecological requirements of forest dependent bats and the response of forest assemblages to different land uses in Asia. Many bat species across South and South‐East Asia, particularly bats in the families Hipposideridae, Rhinolophidae, and the vespertilionid subfamilies Murininae and Kerivoulinae, have wing morphologies and echolocation call types that enable foraging in densely vegetated habitats but which are not effective for foraging in more open habitats (Kingston, 2010; Kingston, Francis, Zubaid, & Kunz, 2003; Meijaard et al., 2005; Wordley et al., 2017). We found that *Rhinolophus beddomei* and *Rhinolophus rouxii* were both significantly more abundant in protected forests than in coffee or forest fragments (Tables 1 and S4); which is expected as they are typical “forest adapted” bats (Wordley et al., 2017). More surprisingly, *Miniopterus pusillus*, which has the long narrow wings and flexible echolocation calls seen in open air foragers (Wordley et al., 2017), was more frequently recorded in protected forest than in forest fragments. This may be because it can forage within the open understorey of protected area forests more easily than in the dense understorey of forest fragments. We saw that several other species were most abundant in protected forests, but they were recorded too infrequently for statistical analysis (Table 1). *Megaderma spasma* was only caught in forest fragments and protected forest and was caught more in protected forest; *Hipposideros pomona* was recorded in all habitats but was most often recorded in protected forests. These species may be the most vulnerable to any land‐use change from protected forest. This underscores the need to examine data beyond richness metrics alone.

Tea is the most heavily modified habitat in the landscape, containing no native bushes or trees. Lower species richness and activity of bats were seen in tea plantations, and bat assemblages were different to those in all other habitats. *M. pusillus* was recorded significantly less frequently in tea plantations, and nine species were not recorded at all. In studies on some taxa, a few species have increased significantly in abundance in intensely modified agricultural landscapes (so‐called winner species; Phalan, Onial, Balmford, & Green, 2011). Both *P. ceylonicus* and *M. fuliginosus* were found more often in tea plantations compared to protected forests, but not compared to the more open habitats of rivers in protected forests. While recorded frequently in tea plantations, these two species have been shown to decline in likelihood of occurrence as the percentage tea cover of an area increases (Wordley et al., 2015). Therefore, tea plantations apparently have no clear “winner” species of bat and many “losers,” echoing the observations of Maas et al. (2015) that there are few “agricultural specialist” bats.

### 4.3 Comparison of riparian habitats

The riparian habitats all tended toward having a greater species richness and activity than their nonriparian counterparts, although the only significant difference was between tea and tea riparian. Species composition also changed between riparian and nonriparian habitats within the agricultural landscape. Other tropical studies reveal that riparian vegetation can be richer in bat species and have higher activity levels than comparable nearby nonriparian vegetation (Monadjem & Reside, 2008; Sirami, Jacobs, & Cumming, 2013; Taylor, Monadjem, & Nicolaas Steyn, 2013), and some bat species show particular preferences for riparian vegetation (Avila‐Cabadilla et al., 2012). Riparian corridors represented a better habitat for bats than rivers through tea with no riparian corridor, but a poorer habitat than rivers in protected areas. The benefits to bats of riparian corridors may be enhanced by having native tree cover on both banks of the river, not just one as seen in this study.

### 4.4 Conservation implications

The high level of protection given to protected forests should be maintained and extended to other intact forests in the Western Ghats. Forest fragments should be maintained and restored for the conservation of bats and other species, by expanding NCF's work in planting native trees in and adjacent to forest fragments to other landscapes in the Ghats. In this landscape, we noted several species which may especially benefit from a focus on restoring forest fragments and riparian corridors. The endangered endemic *Latidens salimalii* was only seen in one riparian corridor and one river in protected forest; this species is severely range restricted and requires conservation measures (Molur & Vanitharani, 2008; Wordley, Foui, Mudappa, Sankaran, & Altringham, 2016). *Megaderma spasma*, while globally widespread, appears sensitive to disturbance as it was never recorded in tea plantations or tea riparian habitats; likewise, *Rhinolophus beddomei* (known only from India and one location in Thailand) and *Rhinolophus rouxii* (largely restricted to South Asia, and one location in Burma) were not recorded in tea plantations and were much rarer outside of protected areas.

Shade coffee under native trees has value in a biodiversity hotspot, but the high value for bats seen in this study may rely on some intact forest remaining in the landscape (Faria & Baumgarten, 2007). There is a growing trend toward the use of non‐native timber trees in coffee plantations in Valparai and globally (Jha et al., 2014); it is important to develop and implement mechanisms that encourage the use of native species, such as premium prices for coffee planters who retain or replant them.

Tea plantations, however, have a significant negative impact on the diversity of all species studied in them thus far (Murali & Raman, 2012; Sidhu et al., 2010; Wordley et al., 2015, 2017). If these plantations are to be made compatible with conservation in a biodiversity hotspot, changes to plantation management are needed. For example, in Valparai, NCF is encouraging tea planters to use native trees for shade rather than the exotic Australian silver oak. Shade will always be sparser for tea than for *Coffea arabica* as tea bushes need more sun, but supplementing the exotic trees with native species may benefit bat diversity. Growers should be rewarded for restoring forest fragments and planting riparian corridors. In areas of high conservation value such as the Western Ghats, it may also be sensible to promote mechanisms to discourage the conversion of shade coffee to tea. The Anamalai Hills were initially planted with shade‐grown coffee (Mudappa & Raman, 2007); it is relatively recently that the landscape has become tea dominated. Localized schemes to reward coffee growers although payments for ecosystem services, access to elite international markets, or help with developing ecotourism could be trialed, alongside or instead of legislation or financial penalties for clearing native trees to plant tea.

While riparian corridors are not equivalent to rivers through protected area forest for bats, they have more value than rivers without riparian corridors. Legislation or incentives to encourage plantation owners to leave a buffer of native trees on each side of every river would greatly benefit bats, and other species in the landscape (Gray, Slade, Mann, & Lewis, 2014; Kumar et al., 2010). The Indian government has committed US$10 billion to planting five million hectares of forest, and improving forest quality on another five million hectares (National Action Plan on Climate Change, 2011). While the main focus of this reforestation drive is for large‐scale work (5,000+ ha plots), riparian corridors in agricultural land may be a good investment for reforestation due to the biodiversity, hydrological, and erosion reducing benefits (Mayer et al., 2007; Sweeney et al., 2004). They may help to restore landscape connectivity and have potential for mitigating human–elephant conflict by providing “migration corridors” through the landscape where elephants can drink, feed, and rest in the shade rather than venturing into tea estates (Kumar et al., 2010). In summary, diverse tropical agricultural landscapes can maintain bat diversity, providing sufficient native trees are maintained.

## CONFLICT OF INTEREST

None declared.

## AUTHOR CONTRIBUTIONS

CFRW was the PhD student on this project and as such conducted the field research and analyses, and wrote most of the manuscript. DM, MS, and JDA contributed to refining ideas for the project, helped with analyses, provided support in field (DM), and edited the manuscript, with JDA as the main PhD supervisor and DM and MS providing in‐country support.
