# Supplementary material for: Heard but not seen: Comparing bat assemblages and study methods in a mosaic landscape in the Western Ghats of India
Source: Ecol Evol. 2018 Mar 23;8(8):3883–94. doi: 10.1002/ece3.3942 (PMC5916271; doi:10.1002/ece3.3942)
Supplement: Supplementary file 1 [file ECE3-8-3883-s001.docx]

Supporting Information

# Table S1: Raw and estimated species richness

| **Both methods combined** | Protected area forest | Forest fragment | Coffee | Tea | Protected area forest river | Riparian corridor | Tea riparian |
| --- | --- | --- | --- | --- | --- | --- | --- |
| Sobs total | 9 | 10 | 10 | 5 | 11 | 10 | 8 |
| Sest total | 9 | 10 | 10 | 5 | 11 | 10 | 8 |
| Sobs mean±S.E. | 7.6±0.51 | 6.4±0.95 | 6.8±0.66 | 3.6±0.4 | 9±0.45 | 6.8±0.58 | 5.8±0.37 |
| Sest mean± S.E. | 8.05±0.5 | 8.5±1.07 | 7.1±0.93 | 3.8±0.5 | 9.6±0.93 | 7±0.63 | 6.3±0.37 |
| **Catching** |  |  |  |  |  |  |  |
| Sobs total | 4 | 3 | 2 | 0 | 6 | 7 | 5 |
| Sest total | 4 | 3 | 2 | 0 | 6 | 7 | 5 |
| Sobs mean± S.E. | 2.6±0.6 | 1.8±0.37 | 1±0.32 | 0±0 | 2.4±0.4 | 1.6±0.87 | 1.8±0.37 |
| Sest mean± S.E. | 3±0.71 | 2±0.55 | 1±0.32 | 0±0 | 2.4±0.4 | 1.6±0.87 | 2.2±0.49 |
| **Acoustic** |  |  |  |  |  |  |  |
| Sobs total | 8 | 7 | 8 | 5 | 10 | 7 | 6 |
| Sest total | 8 | 7 | 8 | 5 | 10 | 7 | 6 |
| Sobs mean± S.E. | 6.4±0.4 | 4.4±0.4 | 6±0.71 | 3.6±0.4 | 8±0.45 | 6±0.45 | 5±0.32 |
| Sest mean± S.E. | 6.93±0.6 | 5±0.32 | 6.2±0.86 | 3.8±0.49 | 8.6±0.93 | 6.4±0.6 | 5.2±0.2 |

# Table S2: Pairwise comparisons of species richness

| **Pairwise comparison of species richness between habitats and methods** | | | | |
| --- | --- | --- | --- | --- |
| **Contrast of habitats and methods. Both = Acoustic and catching methods together, Capture = capture alone, Acoustic = acoustic alone.** | **Estimate**  **±SE** | **Z ratio** | ***P* value** | **Q value (*P* value after FDR correction).** |
| Protected area forest both, Forest fragments both | 0.241±0.164 | 1.466 | 0.143 | 0.210 |
| Protected area forest both, Coffee plantations both | 0.211±0.163 | 1.295 | 0.195 | 0.273 |
| Protected area forest both, Tea plantations both | 0.847±0.199 | 4.253 | <0.0001  *** | <0.0001  *** |
| Protected area forest both, Protected area forest riparian both | -0.154±0.149 | -1.037 | 0.300 | 0.392 |
| Protected area forest both, Riparian corridors both | 0.113±0.159 | 0.713 | 0.476 | 0.555 |
| Protected area forest both, Tea riparian both | 0.288±0.167 | 1.726 | 0.084 | 0.151 |
| Protected area forest both, Protected area forest capture | 1.295±0.142 | 9.107 | <0.0001  *** | <0.0001  *** |
| Protected area forest both, Protected area forest acoustic | 0.155±0.097 | 1.595 | 0.111 | 0.172 |
| Forest fragments both, coffee plantations both | -0.030±0.173 | -0.173 | 0.863 | 0.863 |
| Forest fragments both, Tea plantations both | 0.606±0.207 | 2.925 | 0.003 ** | 0.010  ** |
| Forest fragments both, Protected area forest riparian both | -0.395±0.159 | -2.483 | 0.013 * | 0.029  * |
| Forest fragments both, Riparian corridors both | -0.128±0.169 | -0.757 | 0.449 | 0.546 |
| Forest fragments both, Tea riparian both | 0.047±0.176 | 0.264 | 0.792 | 0.821 |
| Forest fragments both, Forest fragments capture | 1.295±0.142 | 9.107 | <0.0001  *** | <0.0001  *** |
| Forest fragments both, Forest fragments acoustic | 0.155±0.097 | 1.595 | 0.111 | 0.172 |
| Coffee plantations both, Tea plantations both | 0.636±0.206 | 3.086 | 0.002 ** | 0.006  ** |
| Coffee plantations both, Protected area forest riparian both | -0.365±0.158 | -2.316 | 0.021 * | 0.042  * |
| Coffee plantations both, Riparian corridors both | -0.098±0.167 | -0.585 | 0.559 | 0.626 |
| Coffee plantations both, Tea riparian both | 0.076±0.175 | 0.437 | 0.662 | 0.713 |
| Coffee plantations both, Coffee plantations capture | 1.295±0.142 | 9.107 | <0.0001  *** | <0.0001  *** |
| Coffee plantations both, Coffee plantations acoustic | 0.155±0.097 | 1.595 | 0.111 | 0.172 |
| Tea plantations both, Protected area forest riparian both | -1.001±0.195 | -5.139 | <0.0001  *** | <0.0001  *** |
| Tea plantations both, Riparian corridors both | -0.734±0.203 | -3.620 | <0.0001  *** | 0.001  *** |
| Tea plantations both, Tea riparian both | -0.560±0.209 | -2.679 | 0.007 ** | 0.018  * |
| Tea plantations both, Tea plantations capture | 1.295±0.142 | 9.107 | <0.0001  *** | <0.0001  *** |
| Tea plantations both, Tea plantations acoustic | 0.155±0.097 | 1.595 | 0.111 | 0.172 |
| Protected area forest riparian both, Riparian corridors both | 0.267±0.153 | 1.743 | 0.081 | 0.151 |
| Protected area forest riparian both, Tea riparian both | 0.442±0.161 | 2.736 | 0.006 ** | 0.016  * |
| Protected area forest riparian both, Protected area forest riparian capture | 1.295±0.142 | 9.107 | <0.0001  *** | <0.0001  *** |
| Protected area forest riparian both, Protected area forest riparian acoustic | 0.155±0.097 | 1.595 | 0.111 | 0.172 |
| Riparian corridors both, Tea riparian both | 0.174±0.171 | 1.020 | 0.308 | 0.392 |
| Riparian corridors both, Riparian corridors capture | 1.295±0.142 | 9.107 | <0.0001  *** | <0.0001  *** |
| Riparian corridors both, Riparian corridors acoustic | 0.155±0.097 | 1.595 | 0.111 | 0.172 |
| Tea riparian both, Tea riparian capture | 1.295±0.142 | 9.107 | <0.0001  *** | <0.0001  *** |
| Tea riparian both, Tea riparian acoustic | 0.155±0.097 | 1.595 | 0.111 | 0.172 |
| Protected area forest capture, Forest fragments capture | 0.241±0.164 | 1.466 | 0.143 | 0.210 |
| Protected area forest capture, Coffee plantations capture | 0.211±0.163 | 1.295 | 0.195 | 0.273 |
| Protected area forest capture, Tea plantations capture | 0.847±0.199 | 4.253 | <0.0001  *** | <0.0001  *** |
| Protected area forest capture, Protected area forest riparian capture | -0.154±0.149 | -1.037 | 0.300 | 0.392 |
| Protected area forest capture, Riparian corridors capture | 0.113±0.159 | 0.713 | 0.476 | 0.555 |
| Protected area forest capture, Tea riparian capture | 0.288±0.167 | 1.726 | 0.084 | 0.151 |
| Protected area forest capture, Protected area forest acoustic | -1.140±0.145 | -7.877 | <0.0001  *** | <0.0001  *** |
| Forest fragments capture, Coffee plantations capture | -0.030±0.173 | -0.173 | 0.863 | 0.863 |
| Forest fragments capture, Tea plantations capture | 0.606±0.207 | 2.925 | 0.003 ** | 0.010  ** |
| Forest fragments capture, Protected area forest riparian capture | -0.395±0.159 | -2.483 | 0.013 * | 0.029  * |
| Forest fragments capture, Riparian corridors capture | -0.128±0.169 | -0.757 | 0.449 | 0.546 |
| Forest fragments capture, Tea riparian capture | 0.047±0.176 | 0.264 | 0.792 | 0.821 |
| Forest fragments capture, Forest fragments acoustic | -1.140±0.145 | -7.877 | <0.0001  *** | <0.0001  *** |
| Coffee plantations capture, Tea plantations capture | 0.636±0.206 | 3.086 | 0.002 ** | 0.006  ** |
| Coffee plantations capture, Protected area forest riparian capture | -0.365±0.158 | -2.316 | 0.021 * | 0.042  * |
| Coffee plantations capture, Riparian corridors capture | -0.098±0.167 | -0.585 | 0.559 | 0.626 |
| Coffee plantations capture, Tea riparian capture | 0.076±0.175 | 0.437 | 0.662 | 0.713 |
| Coffee plantations capture, Coffee plantations acoustic | -1.140±0.145 | -7.877 | <0.0001  *** | <0.0001  *** |
| Tea plantations capture, Protected area forest riparian capture | -1.001±0.195 | -5.139 | <0.0001  *** | <0.0001  *** |
| Tea plantations capture, Riparian corridors capture | -0.734±0.203 | -3.620 | <0.0001  *** | 0.001  *** |
| Tea plantations capture, Tea riparian capture | -0.560±0.209 | -2.679 | 0.007 ** | 0.018  * |
| Tea plantations capture, Tea plantations acoustic | -1.140±0.145 | -7.877 | <0.0001  *** | <0.0001  *** |
| Protected area forest riparian capture, Riparian corridors capture | 0.267±0.153 | 1.743 | 0.081 | 0.151 |
| Protected area forest riparian capture, Tea riparian capture | 0.442±0.161 | 2.736 | 0.006 ** | 0.016  * |
| Protected area forest riparian capture, Protected area forest riparian acoustic | -1.140±0.145 | -7.877 | <0.0001  *** | <0.0001  *** |
| Riparian corridors capture, Tea riparian capture | 0.174±0.171 | 1.020 | 0.308 | 0.392 |
| Riparian corridors capture, Riparian corridors acoustic | -1.140±0.145 | -7.877 | <0.0001  *** | <0.0001  *** |
| Tea riparian capture, Tea riparian acoustic | -1.140±0.145 | -7.877 | <0.0001  *** | <0.0001  *** |
| Protected area forest acoustic, Forest fragments acoustic | 0.241±0.164 | 1.466 | 0.143 | 0.210 |
| Protected area forest acoustic, Coffee plantations acoustic | 0.211±0.163 | 1.295 | 0.195 | 0.273 |
| Protected area forest acoustic, Tea plantations acoustic | 0.847±0.199 | 4.253 | <0.0001  *** | <0.0001  *** |
| Protected area forest acoustic, Protected area forest riparian acoustic | -0.154±0.149 | -1.037 | 0.300 | 0.392 |
| Protected area forest acoustic, Riparian corridors acoustic | 0.113±0.159 | 0.713 | 0.476 | 0.555 |
| Protected area forest acoustic, Tea riparian acoustic | 0.288±0.167 | 1.726 | 0.084 | 0.151 |
| Forest fragments acoustic, Coffee plantations acoustic | -0.030±0.173 | -0.173 | 0.863 | 0.863 |
| Forest fragments acoustic, Tea plantations acoustic | 0.606±0.207 | 2.925 | 0.003 ** | 0.010  ** |
| Forest fragments acoustic, Protected area forest riparian acoustic | -0.395±0.159 | -2.483 | 0.013 * | 0.029  * |
| Forest fragments acoustic, Riparian corridors acoustic | -0.128±0.169 | -0.757 | 0.449 | 0.546 |
| Forest fragments acoustic, Tea riparian acoustic | 0.047±0.176 | 0.264 | 0.792 | 0.821 |
| Coffee plantations acoustic, Tea plantations acoustic | 0.636±0.206 | 3.086 | 0.002 ** | 0.006  ** |
| Coffee plantations acoustic, Protected area forest riparian acoustic | -0.365±0.158 | -2.316 | 0.021 * | 0.042  * |
| Coffee plantations acoustic, Riparian corridors acoustic | -0.098±0.167 | -0.585 | 0.559 | 0.626 |
| Coffee plantations acoustic, Tea riparian acoustic | 0.076±0.175 | 0.437 | 0.662 | 0.713 |
| Tea plantations acoustic, Protected area forest riparian acoustic | -1.001±0.195 | -5.139 | <0.0001  *** | <0.0001  *** |
| Tea plantations acoustic, Riparian corridors acoustic | -0.734±0.203 | -3.620 | <0.0001  *** | 0.001  *** |
| Tea plantations acoustic, Tea riparian acoustic | -0.560±0.209 | -2.679 | 0.007 ** | 0.018  * |
| Protected area forest riparian acoustic, Riparian corridors acoustic | 0.267±0.153 | 1.743 | 0.081 | 0.151 |
| Protected area forest riparian acoustic, Tea riparian acoustic | 0.442±0.161 | 2.736 | 0.006 **± | 0.016  * |
| Riparian corridors acoustic, Tea riparian acoustic | 0.174±0.171 | 1.020 | 0.308 | 0.392 |

# Table S3: Pairwise comparisons of activity

|  | **Pairwise comparison of activity between habitats and methods** | | | | |
| --- | --- | --- | --- | --- | --- |
| **Contrast of habitats and methods. Both = acoustic and capture methods together, Capture = capture alone, Acoustic = acoustic alone.** | | **Estimate**  **±SE** | **Z ratio** | ***P* value** | **Q value (*P* value after FDR correction).** |
| Protected area forest both, Forest fragments both | | 0.693±0.19 | 3.657 | <0.0001  *** | 0.001 *** |
| Protected area forest both, Coffee plantations both | | 0.081±0.158 | 0.51 | 0.61 | 0.657 |
| Protected area forest both, Tea plantations both | | 0.670±0.188 | 3.56 | <0.0001  *** | 0.001 *** |
| Protected area forest both, Protected area forest riparian both | | -0.274±0.145 | -1.888 | 0.059 | 0.081 |
| Protected area forest both, Riparian corridors both | | -0.086±0.152 | -0.564 | 0.573 | 0.657 |
| Protected area forest both, Tea riparian both | | 0.081±0.158 | 0.51 | 0.61 | 0.657 |
| Protected area forest both, Protected area forest capture | | 1.560±0.148 | 10.511 | <0.0001  *** | <0.0001  *** |
| Protected area forest both, Protected area forest acoustic | | 0.236±0.093 | 2.533 | 0.011 * | 0.018 * |
| Forest fragments both, coffee plantations both | | -0.613±0.192 | -3.188 | 0.001 *** | 0.003 ** |
| Forest fragments both, Tea plantations both | | -0.024±0.218 | -0.108 | 0.914 | 0.948 |
| Forest fragments both, Protected area forest riparian both | | -0.967±0.182 | -5.320 | <0.0001  *** | <0.0001  *** |
| Forest fragments both, Riparian corridors both | | -0.779±0.187 | -4.165 | <0.0001  *** | <0.0001  *** |
| Forest fragments both, Tea riparian both | | -0.613±0.192 | -3.188 | 0.001 *** | 0.003 ** |
| Forest fragments both, Forest fragments capture | | 1.56±0.148 | 10.511 | <0.0001  *** | <0.0001  *** |
| Forest fragments both, Forest fragments acoustic | | 0.236±0.093 | 2.533 | 0.011 * | 0.018 * |
| Coffee plantations both, Tea plantations both | | 0.589±0.191 | 3.088 | 0.002 ** | 0.004 ** |
| Coffee plantations both, Protected area forest riparian both | | -0.355±0.149 | -2.387 | 0.017 * | 0.025 * |
| Coffee plantations both, Riparian corridors both | | -0.166±0.155 | -1.072 | 0.284 | 0.345 |
| Coffee plantations both, Tea riparian both | | 0.000±0.161 | 0.000 | 1 | 1 |
| Coffee plantations both, Coffee plantations capture | | 1.560±0.148 | 10.511 | <0.0001  *** | <0.0001  *** |
| Coffee plantations both, Coffee plantations acoustic | | 0.236±0.093 | 2.533 | 0.011 * | 0.018 * |
| Tea plantations both, Protected area forest riparian both | | -0.944±0.18 | -5.235 | <0.0001  *** | <0.0001  *** |
| Tea plantations both, Riparian corridors both | | -0.755±0.185 | -4.072 | <0.0001  *** | <0.0001  *** |
| Tea plantations both, Tea riparian both | | -0.589±0.191 | -3.088 | 0.002 ** | 0.004 ** |
| Tea plantations both, Tea plantations capture | | 1.560±0.148 | 10.511 | <0.0001  *** | <0.0001  *** |
| Tea plantations both, Tea plantations acoustic | | 0.236±0.093 | 2.533 | 0.011 * | 0.018 * |
| Protected area forest riparian both, Riparian corridors both | | 0.189±0.142 | 1.331 | 0.183 | 0.241 |
| Protected area forest riparian both, Tea riparian both | | 0.355±0.149 | 2.387 | 0.017 * | 0.025 * |
| Protected area forest riparian both, Protected area forest riparian capture | | 1.560±0.148 | 10.511 | <0.0001  *** | <0.0001  *** |
| Protected area forest riparian both, Protected area forest riparian acoustic | | 0.236±0.093 | 2.533 | 0.011 * | 0.018 * |
| Riparian corridors both, Tea riparian both | | 0.166±0.155 | 1.072 | 0.284 | 0.345 |
| Riparian corridors both, Riparian corridors capture | | 1.560±0.148 | 10.511 | <0.0001  *** | <0.0001  *** |
| Riparian corridors both, Riparian corridors acoustic | | 0.236  ±0.093 | 2.533 | 0.011 * | 0.018 * |
| Tea riparian both, Tea riparian capture | | 1.56±0.148 | 10.511 | <0.0001  *** | <0.0001  *** |
| Tea riparian both, Tea riparian acoustic | | 0.236±0.093 | 2.533 | 0.011 * | 0.018 * |
| Protected area forest capture, Forest fragments capture | | 0.693±0.19 | 3.657 | <0.0001  *** | 0.001 *** |
| Protected area forest capture, Coffee plantations capture | | 0.081±0.158 | 0.510 | 0.61 | 0.657 |
| Protected area forest capture, Tea plantations capture | | 0.67±0.188 | 3.560 | <0.0001  *** | 0.001 *** |
| Protected area forest capture, Protected area forest riparian capture | | -0.274±0.145 | -1.888 | 0.059 | 0.081 |
| Protected area forest capture, Riparian corridors capture | | -0.086±0.152 | -0.564 | 0.573 | 0.657 |
| Protected area forest capture, Tea riparian capture | | 0.081±0.158 | 0.510 | 0.61 | 0.657 |
| Protected area forest capture, Protected area forest acoustic | | -1.324±0.152 | -8.723 | <0.0001  *** | <0.0001  *** |
| Forest fragments capture, Coffee plantations capture | | -0.613±0.192 | -3.188 | 0.001 *** | 0.003 ** |
| Forest fragments capture, Tea plantations capture | | -0.024±0.218 | -0.108 | 0.914 | 0.948 |
| Forest fragments capture, Protected area forest riparian capture | | -0.967±0.182 | -5.320 | <0.0001  *** | <0.0001  *** |
| Forest fragments capture, Riparian corridors capture | | -0.779±0.187 | -4.165 | <0.0001  *** | <0.0001  *** |
| Forest fragments capture, Tea riparian capture | | -0.613±0.192 | -3.188 | 0.001 *** | 0.003 ** |
| Forest fragments capture, Forest fragments acoustic | | -1.324±0.152 | -8.723 | <0.0001  *** | <0.0001  *** |
| Coffee plantations capture, Tea plantations capture | | 0.589±0.191 | 3.088 | 0.002 ** | 0.004 ** |
| Coffee plantations capture, Protected area forest riparian capture | | -0.355±0.149 | -2.387 | 0.017 * | 0.025 * |
| Coffee plantations capture, Riparian corridors capture | | -0.166±0.155 | -1.072 | 0.284 | 0.345 |
| Coffee plantations capture, Tea riparian capture | | 0±0.161 | 0 | 1 | 1 |
| Coffee plantations capture, Coffee plantations acoustic | | -1.405±0.219 | -6.412 | <0.0001  *** | <0.0001  *** |
| Tea plantations capture, Protected area forest riparian capture | | -1.324±0.152 | -8.723 | <0.0001  *** | <0.0001  *** |
| Tea plantations capture, Riparian corridors capture | | -0.944±0.180 | -5.235 | <0.0001  *** | <0.0001  *** |
| Tea plantations capture, Tea riparian capture | | -0.755±0.185 | -4.072 | <0.0001  *** | <0.0001  *** |
| Tea plantations capture, Tea plantations acoustic | | -0.589±0.191 | -3.088 | 0.002 ** | 0.004 ** |
| Protected area forest riparian capture, Riparian corridors capture | | -1.324±0.152 | -8.723 | <0.0001  *** | <0.0001  *** |
| Protected area forest riparian capture, Tea riparian capture | | 0.189±0.142 | 1.331 | 0.183 | 0.241 |
| Protected area forest riparian capture, Protected area forest riparian acoustic | | 0.355±0.149 | 2.387 | 0.017 * | 0.025 * |
| Riparian corridors capture, Tea riparian capture | | -1.324±0.152 | -8.723 | <0.0001  *** | <0.0001  *** |
| Riparian corridors capture, Riparian corridors acoustic | | -1.324±0.152 | -8.723 | <0.0001  *** | <0.0001  *** |
| Tea riparian capture, Tea riparian acoustic | | -1.324±0.152 | -8.723 | <0.0001  *** | <0.0001  *** |
| Protected area forest acoustic, Forest fragments acoustic | | 0.693±0.19 | 3.657 | <0.0001  *** | 0.001 *** |
| Protected area forest acoustic, Coffee plantations acoustic | | 0.081±0.158 | 0.510 | 0.61 | 0.657 |
| Protected area forest acoustic, Tea plantations acoustic | | 0.67±0.188 | 3.560 | <0.0001  *** | 0.001 *** |
| Protected area forest acoustic, Protected area forest riparian acoustic | | -0.274±0.145 | -1.888 | 0.059 | 0.081 |
| Protected area forest acoustic, Riparian corridors acoustic | | -0.086±0.152 | -0.564 | 0.573 | 0.657 |
| Protected area forest acoustic, Tea riparian acoustic | | 0.081±0.158 | 0.510 | 0.61 | 0.657 |
| Forest fragments acoustic, Coffee plantations acoustic | | -0.613±0.192 | -3.188 | 0.001 *** | 0.003 ** |
| Forest fragments acoustic, Tea plantations acoustic | | -0.024±0.218 | -0.108 | 0.914 | 0.948 |
| Forest fragments acoustic, Protected area forest riparian acoustic | | -0.967±0.182 | -5.320 | <0.0001  *** | <0.0001  *** |
| Forest fragments acoustic, Riparian corridors acoustic | | -0.779±0.187 | -4.165 | <0.0001  *** | <0.0001  *** |
| Forest fragments acoustic, Tea riparian acoustic | | -0.613±0.192 | -3.188 | 0.001 *** | 0.003 ** |
| Coffee plantations acoustic, Tea plantations acoustic | | 0.589±0.191 | 3.088 | 0.002 ** | 0.004 ** |
| Coffee plantations acoustic, Protected area forest riparian acoustic | | -0.355±0.149 | -2.387 | 0.017 * | 0.025 * |
| Coffee plantations acoustic, Riparian corridors acoustic | | -0.166±0.155 | -1.072 | 0.284 | 0.345 |
| Coffee plantations acoustic, Tea riparian acoustic | | 0±0.161 | 0.000 | 1 | 1 |
| Tea plantations acoustic, Protected area forest riparian acoustic | | -0.944±0.180 | -5.235 | <0.0001  *** | <0.0001  *** |
| Tea plantations acoustic, Riparian corridors acoustic | | -0.755±0.185 | -4.072 | <0.0001  *** | <0.0001  *** |
| Tea plantations acoustic, Tea riparian acoustic | | -0.589±0.191 | -3.088 | 0.002 ** | 0.004 ** |
| Protected area forest riparian acoustic, Riparian corridors acoustic | | 0.189±0.142 | 1.331 | 0.183 | 0.241 |
| Protected area forest riparian acoustic, Tea riparian acoustic | | 0.355±0.149 | 2.387 | 0.017 * | 0.025 * |
| Riparian corridors acoustic, Tea riparian acoustic | | 0.166±0.155 | 1.072 | 0.284 | 0.345 |

# Table S4: Changes in species abundance

| **Mean number of records per habitat (using both methods combined) with standard deviations and Kruskal-Wallis comparisons of abundances, for species with >30 individuals. Letters indicate differences in FDR adjusted p values of pairwise comparisons between habitats – habitats with the same letter are not-significantly different, habitats with different letters are significantly different a P<0.05. <0.05=*, <0.01=**, <0.001=***** | | | | | | | | | | |
| --- | --- | --- | --- | --- | --- | --- | --- | --- | --- | --- |
| **Species** | **Protected area forest mean ± se** | **Forest fragment mean ± se** | **Coffee mean ± se** | **Tea mean ± se** | **Protected area forest river mean ± se** | **Riparian corridor mean ± se** | **Tea riparian mean ± se** | **χ2** | **df** | ***P* value** |
| *Cynopterus brachyotis* | 4.40±2.62  ab | 4.4±1.44  ab | 5.8±3.26  ab | 0±0  c | 6.20±1.83  a | 2.6±1.65  bc | 0±0  c | 16.63 | 6 | 0.0107 * |
| *Myotis horsfieldii* | 0±0  b | 0±0  b | 0±0  b | 0±0  b | 5.2±1.32  a | 5.6±2.24  a | 5.2±1.98  a | 25.13 | 6 | 0.0003 *** |
| *Miniopterus fuliginosus* | 2.2±1.02  b | 1.6±0.4  b | 5.8±0.86  a | 6.4±1.4  a | 4.6±2.07  ab | 5.6±1.67  a | 6±0.77  a | 14.78 | 6 | 0.022 * |
| *Miniopterus pusillus* | 5.4±2.07  ab | 1±0.63  c | 4±1.05  ab | 1.6±0.81  bc | 7±1.73  a | 2.4±0.51  abc | 1.8±0.66  bc | 11.77 | 6 | 0.067 |
| *Pipistrellus ceylonicus* | 4.2±1.16  de | 4±0.95  e | 7.2±1.32  c | 7.6±0.93  bc | 7.2±0.58  cd | 10.4±1.21  ab | 11±0.95  a | 21.54 | 6 | 0.0015 ** |
| *Rhinolophus beddomei* | 3.2±0.97  a | 0.4±0.4  b | 0.4±0.4  b | 0±0  b | 2.4±0.51  a | 0.2±0.21  b | 0±0  b | 24.82 | 6 | 0.0004 *** |
| *Rhinolophus indorouxii* | 1±0.63 | 1.6±0.68 | 1.8±1.07 | 0.6±0.25 | 0.8±0.8 | 2.8±1.2 | 0.8±0.58 | 5.79 | 6 | 0.447 |
| *Rhinolophus lepidus* | 3.4±1.03  bc | 2.4±0.93  bc | 4.2±0.38  ab | 1±0.63  c | 6.8±1.32  a | 5±1.23  ab | 4.4±1.21  ab | 14.19 | 6 | 0.0276 * |
| *Rhinolophus rouxii* | 6.8±2.94  a | 0.±0.6  bc | 0.4±0.4  bc | 0±0  c | 1.8±0.97  ab | 0±0  c | 0±0  c | 12.85 | 6 | 0.046 |

# Table S5: Differences in species composition, capture only

| **ADONIS differences in species composition using capture methods with false discovery rate (FDR) corrections.** | | | | |
| --- | --- | --- | --- | --- |
| **Compare** | **Against** | **F** | ***P* value** | **Q value (after FDR correction)** |
| Coffee | Forest fragments | 1.922 | 0.055 | 0.111 |
| Coffee | Riparian corridors | NaN | NA | NA |
| Coffee | Tea riparian | 2.080 | 0.089 | 0.124 |
| Coffee | Tea | NaN | NA | NA |
| Coffee | Protected area forest | 1.204 | 0.307 | 0.322 |
| Coffee | Protected area forest riparian | 1.165 | 0.322 | 0.322 |
| Forest fragments | Riparian corridors | 2.583 | 0.026 * | 0.070 |
| Forest fragments | Tea riparian | 7.273 | 0.008 ** | 0.034 * |
| Forest fragments | Tea | NaN | NA | NA |
| Forest fragments | Protected area forest | 3.772 | 0.030 * | 0.070 |
| Forest fragments | Protected area forest riparian | 2.350 | 0.075 | 0.124 |
| Riparian corridors | Tea riparian | 1.857 | 0.007 ** | 0.034 * |
| Riparian corridors | Tea | NaN | NA | NA |
| Riparian corridors | Protected area forest | 1.226 | 0.305 | 0.322 |
| Riparian corridors | Protected area forest riparian | 1.671 | 0.110 | 0.140 |
| Tea riparian | Tea | NaN | NA | NA |
| Tea riparian | Protected area forest | 3.595 | 0.006 ** | 0.034 * |
| Tea riparian | Protected area forest riparian | 5.479 | 0.010 ** | 0.034 * |
| Tea | Protected area forest | NaN | NA | NA |
| Tea | Protected area forest riparian | NaN | NA | NA |
| Protected area forest | Protected area forest riparian | 2.269 | 0.088 | 0.124 |

# Table S6: Differences in species composition, acoustic only

| **ADONIS differences in species composition using acoustic methods with false discovery rate (FDR) corrections.** | | | | |
| --- | --- | --- | --- | --- |
| **Compare** | **Against** | **F** | ***P* value** | **Q value (after FDR correction)** |
| Coffee | Forest fragments | 3.946 | 0.010 ** | 0.019 * |
| Coffee | Riparian corridors | 2.556 | 0.065 | 0.072 |
| Coffee | Tea riparian | 3.130 | 0.008 ** | 0.018 * |
| Coffee | Tea | 2.794 | 0.009 ** | 0.018 * |
| Coffee | Protected area forest | 2.478 | 0.050 * | 0.058 |
| Coffee | Protected area forest riparian | 4.551 | 0.009 ** | 0.018 * |
| Forest fragments | Riparian corridors | 4.650 | 0.008 ** | 0.018 * |
| Forest fragments | Tea riparian | 5.555 | 0.008 ** | 0.018 * |
| Forest fragments | Tea | 3.360 | 0.033 * | 0.046 * |
| Forest fragments | Protected area forest | 1.758 | 0.142 | 0.149 |
| Forest fragments | Protected area forest riparian | 6.206 | 0.008 ** | 0.018 * |
| Riparian corridors | Tea riparian | 0.128 | 0.913 | 0.913 |
| Riparian corridors | Tea | 3.732 | 0.007 ** | 0.018 * |
| Riparian corridors | Protected area forest | 4.368 | 0.008 ** | 0.018 * |
| Riparian corridors | Protected area forest riparian | 3.277 | 0.024 * | 0.035 * |
| Tea riparian | Tea | 3.190 | 0.041 * | 0.054 |
| Tea riparian | Protected area forest | 5.310 | 0.008 ** | 0.018 * |
| Tea riparian | Protected area forest riparian | 4.775 | 0.017 * | 0.027 * |
| Tea | Protected area forest | 4.407 | 0.016 * | 0.027 * |
| Tea | Protected area forest riparian | 9.039 | 0.009 ** | 0.018 * |
| Protected area forest | Protected area forest riparian | 2.284 | 0.048 * | 0.058 |

# Table S7: Size versus richness

| Habitat | Site | Size (ha) | Total Richness |
| --- | --- | --- | --- |
| Forest fragment | Tata Finlay | 37.5 | 9 |
| Forest fragment | Selaliparai | 2.2 | 7 |
| Forest fragment | Varattuparai 2,3 | 14.4 | 7 |
| Forest fragment | Sangli Road | 102.8 | 7 |
| Forest fragment | Iyerpadi Top | 78.5 | 7 |
| Riparian corridor | Iyerpadi riparian corridor | 3.7 | 7 |
| Riparian corridor | Kududarai riparian corridor | 20.5 | 6 |
| Riparian corridor | Gajamudi riparian corridor | 20.5 | 10 |
| Riparian corridor | Sholayar Dam riparian corridor | 159.7 | 7 |
| Riparian corridor | Sangli Road riparian corridor | 102.8 | 11 |
